# Impact of a short-term nitrate and citrulline co-supplementation on sport performance in elite rowers: a randomized, double-blind, placebo-controlled crossover trial

**DOI:** 10.1007/s00421-024-05415-4

**Published:** 2024-02-10

**Authors:** Aitor Viribay, Juan M. A. Alcantara, Iker López, Juan Mielgo-Ayuso, Arkaitz Castañeda-Babarro

**Affiliations:** 1 Glut4Science, Physiology, Nutrition and Sport, 01004 Vitoria-Gasteiz, Spain; 2 Institute of Biomedicine (IBIOMED), University of Leon, 24071 Leon, Spain; 3 Department of Health Sciences, Institute for Sustainability and Food Chain Innovation, Public University of Navarre, Pamplona, Spain; 4 Navarra Institute for Health Research, IdiSNA, Pamplona, Spain; 5 Centro de Investigación Biomédica en Red Fisiopatología de la Obesidad y Nutrición (CIBERobn), Instituto de Salud Carlos III, 28029 Madrid, Spain; 6 Kirolene, San Ignacio Auzunea Etxetaldea 5, 48200 Durango, Spain; 7 Department of Health Sciences, Faculty of Health Sciences, University of Burgos, 09001 Burgos, Spain; 8 Health, Physical Activity, and Sports Science Laboratory, Department of Physical Activity and Sports, Faculty of Education and Sport, University of Deusto, 48007 Bizkaia, Spain

**Keywords:** Physiology, Oxide nitric, Supplementation, Metabolism

## Abstract

**Purpose:**

Citrulline (CIT) and beetroot extract (BR) have separately shown benefits in rowing performance-related outcomes. However, effects of combined supplementation remain to be elucidated. The main purpose of this research was to study the effects of 1 week of daily co-supplementation of 3.5 g BR (500 mg NO_3_^−^) plus 6 g CIT on aerobic performance, maximal strength, and high-intensity power and peak stroke in elite male rowers compared to a placebo and to a BR supplementation.

**Methods:**

20 elite rowers participated in this randomized, double-blind, placebo-controlled crossover trial completing 1 week of supplementation in each group of study: Placebo group (PLAG); BR group (BRG); and BR + CIT group (BR-CITG). 3 main physical tests were performed: aerobic performance, Wingate test and CMJ jump, and metabolic biomarkers and physiological outcomes were collected.

**Results:**

The Wingate all-out test showed no between-condition differences in peak power, mean power, relative power, or fatigue index (*P* > 0.05), but clearance of lactate was better in BR-CITG (*P* < 0.05). In the performance test, peak power differed only between PLAG and BR-CITG (*P* = 0.036), while VO2peak and maximum heart rate remained similar. CMJ jumping test results showed no between-condition differences, and blood samples were consistent (*P* > 0.200).

**Conclusion:**

Supplementation with 3.5 g of BR extract plus 6 g of CIT for 7 days improved lactate clearance after Wingate test and peak power in a performance test. No further improvements were found, suggesting longer period of supplementation might be needed to show greater benefits.

**Supplementary Information:**

The online version contains supplementary material available at 10.1007/s00421-024-05415-4.

## Introduction

Aerobic performance and maximal power have been proposed as the 2 main physiological determinants and predictors of rowing performance (Ingham et al. 2002; Izquierdo-Gabarren et al. 2010). Aerobic performance can be explained by central and peripheral factors determining the capacity to sustain power production for a given time (Coyle 2007). Among them, whole-body peak oxygen uptake (VO_2peak_) is a widely used outcome associated with performance and exercise capacity and determines the aerobic capacity of an individual (Bassett and Howley 2000). In professional rowers, the VO_2peak_ is positively correlated with performance during high-intensity endurance efforts lasting between 5 and 7 min (Papadakis et al. 2022; Penichet-Tomas et al. 2023). VO_2peak_ is partly determined by the capacity of the heart to provide blood to peripheral tissues—explained by the stroke volume and maximal heart rate—and therefore, maximum heart rate has been always considered a critical outcome (Coyle 2007). Moreover, aerobic performance can be also explained by the ability to oxidize substrates at cellular level—the so-called mitochondrial function—that is commonly assessed in sports field by the blood lactate concentration due to its inverse correlation with fatty acid oxidation and mitochondrial respiration (San-Millán and Brooks 2018; Brooks 2020). In addition, the ability to produce and maintain peak stroke power is positively associated with muscle strength (maximal strength) (Bell et al. 1989) and related to performance (the higher is the power production—high peak power—and maintenance—high mean power and low fatigue index—, the higher the rowing performance) (Lawton et al. 2013).

Both main physiological determinants are usually improved with training and nutritional interventions and optimized to a greater level with additional interventions (i.e., use of supplementation products) that athletes rely on (Jeukendrup 2017; Tiller et al. 2019). Nitric oxide synthase-related supplements such as nitrate-rich beetroot (BR) extract, or amino acids such as citrulline (CIT) and arginine, have garnered attention due to their potential beneficial influence on outcomes related to performance (Bescos et al. 2012; Bryan 2018; Maughan et al. 2018). Nitrate serves as the precursor of nitric oxide (NO), an important bioactive molecule that is produced endogenously from L-arginine, as extensively described elsewhere (Lundberg et al. 2008; Milton-Laskibar et al. 2021). Briefly, NO production from precursors’ supplementation produced positive effects that could positively influence sports performance (Bailey et al. 2009; Bescos et al. 2012; Jones 2014a; Dominguez et al. 2018; Gonzalez and Trexler 2020; Viribay et al. 2020).

Supplementation with BR elevates circulating nitrate (NO_3_^−^) levels and the production of NO (Milton-Laskibar et al. 2021). BR’s most remarkable effects are vascular vasodilation, increasing blood flow supply and tissue oxygenation (Clements et al. 2014), and acute or chronic BR supplementation has shown positive effects on performance. Previous literature showed that BR ingested 2.5 h before exercise improved aerobic power by reducing the total time to complete a 4 km time trial in cyclists (Lausch et al. 2017). Similar effects were observed in highly trained rowers ingesting this supplement 2 h before exercise (Hoon et al. 2014). Concerning short-term ingestion (8 days of daily BR intake), a previous study showed no improvement on a 1500 m time trial in elite runners (Boorsma et al. 2014). Conversely, in a sample of trained rowers, 6 days of daily BR intake improved exercise performance after consecutive 6 × 500 m trials (Bond et al. 2012). In addition, BR seems to improve human muscle contractile capacity (Coggan and Peterson 2018; Coggan et al. 2021) and could positively influence muscle strength and peak force production. A previous study performed in trained adolescents showed improvements in all-out Wingate test-related parameters such as peak power, average power, and fatigue index after BR intake 2.5 h before exercising (Bender et al. 2018). Daily supplementation of BR for 6 days improved resistance-trained males’ strength and resistance exercise performance (Mosher et al. 2016). Nevertheless, differences between studies are large and that could be related to the amount of BR intake (BR amount ranged from 5.5 to 19.7 mmol) which arouses the necessity to study the potential correlation between effects and supplementation dosage (Bond et al. 2012; Hoon et al. 2014; Boorsma et al. 2014; Mosher et al. 2016; Lausch et al. 2017; Coggan and Peterson 2018; Bender et al. 2018; Coggan et al. 2021). The CIT is an amino acid that possesses potential ergogenic properties, as extensively described elsewhere (Curis et al. 2005; Wu et al. 2009). CIT supplementation is supposed to reduce the accumulation of blood lactate and acidosis during high-intensity exercise, which may result in enhanced aerobic performance and muscle strength production (Rhim et al. 2020). In trained males, CIT consumed 1 h before exercise reducing the time necessary to cover 4 km (Suzuki et al. 2016). Similarly, in healthy males and females, CIT consumed 3 h before exercise increasing the time until exhaustion (Hickner et al. 2006). Concerning muscle strength production, CIT supplementation could stimulate muscle protein synthesis (Goron et al. 2019). For instance, acute ingestion of CIT 1 h pre-exercise favored improvements in the strength production capacity in both trained and untrained males (Pérez-Guisado and Jakeman 2010; Wax et al. 2015; Trexler et al. 2019). Despite the potential effects of aforementioned studies, two recent systematic review and meta-analysis studies suggested that CIT supplementation might not influence aerobic exercise performance (Viribay et al. 2022) and muscle strength production capacity (Aguiar and Casonatto 2022). However, a wide range of intensities (60–100% VO_2max_) were included in one meta-analysis (Viribay et al. 2022) and only 4 studies were included in the remaining one (Aguiar and Casonatto 2022), suggesting data around effects of CIT on VO_2peak_ and strength is still scarce. Nevertheless, as BR and CIT supplements elevate NO production (Bescos et al. 2012), the study of their ergogenic effects on athletes’ performance deserves attention (Lansley et al. 2011; Hernández et al. 2012; Haider and Folland 2014). Nowadays, an emerging field of study is the combination of BR and CIT supplements. In fact, it is plausible to hint at potential synergic benefits with the combination of BR and CIT (Roux-Mallouf et al. 2019), and other NO-related supplements such as CIT and arginine (Suzuki et al. 2019). In this regard, improvements in aerobic power performance and maximal and endurance strength have been found after 9 weeks of combined supplementation in trained miles (Burgos et al. 2021). Moreover, same supplementation protocol confirmed enhanced recovery status (biochemical parameters) and performance in aerobic power test (Burgos et al. 2022). However, available literature on the combined supplementation of BR and CIT using different intake durations (e.g., long-time intake supplementation vs. acute intake supplementation), and across sport modalities is still limited. Thus, their potential effect on performance deserves attention.

The main objective of this study was to determine the effects of short-term intervention (1 week) with oral co-supplementation of BR (3.5 g per day) extract (500 mg NO_3_^−^ per day) plus CIT (6 g per day) on aerobic performance (power output, VO_2 peak_, lactate clearance and heart rate), by graded incremental test, maximal strength by counter-movement jump test (height), and high-intensity power and peak stroke by Wingate test (mean, relative and peak power, and fatigue index) in elite male rowers compared to a placebo and to a BR supplementation (1 week each).

## Methods

### Study design and participants

**Fig. 1:**
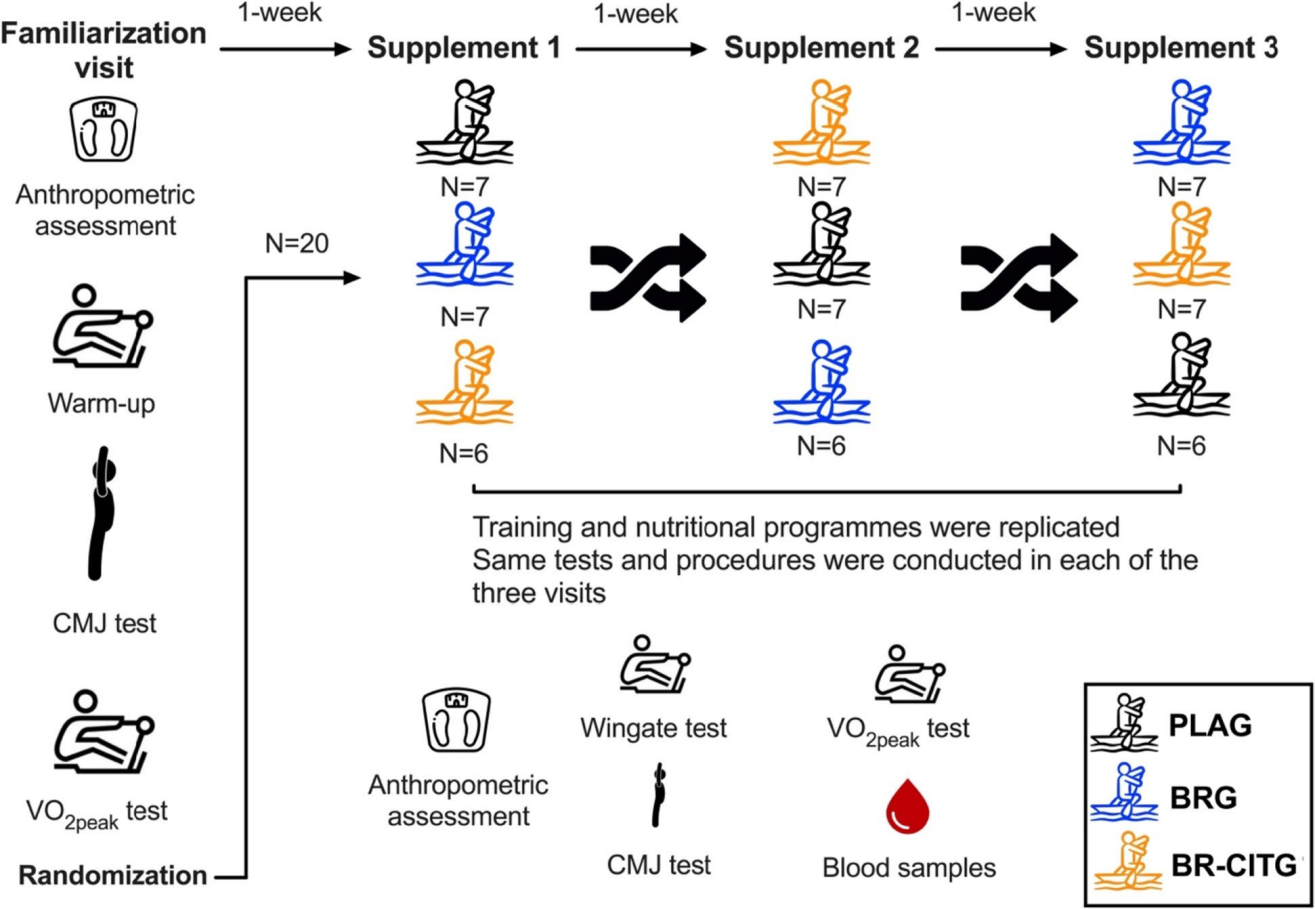
Study design detailing the familiarization visit and the three study visits (replicated) after every week of supplementation. *CMJ* Countermovement jump test, *VO*_*2peak*_ whole-body peak oxygen uptake, *PLAG* placebo supplement (maltodextrin) group, *BRG* nitrate-rich beetroot extract supplement group, *BR-CITG* BR extract plus citrulline (CIT) supplement group

Twenty male elite rowers from the first rowing professional Spanish league Asociación de Clubes de Traineras (ACT) with an average of 14 years of experience in rowing participated in this randomized, double-blind, placebo-controlled crossover trial (Fig. 1). Initially, the rowers were evaluated by a researcher with a precise anamnesis and medical examination. The rowers were not on any prescribed medication and were not following any nutritional intervention (e.g., supplementation and/or ergogenic aids) or dietary advice before their inclusion in the current study.

The rowers trained a total of 10 h/week, with a frequency of 6 days/week and internal training load was monitored using a heart rate monitor and by asking athletes’ rate of perceived effort (Borg RPE-CR10 scale). Their outdoor training routine involved both, aerobic and specific outdoor rowing training (5 h/session; 2 sessions/week; 70–80% maximum heart rate). During their indoor training routine, the rowers elicited in a rowing ergometer (3 h/session; 2 sessions/week; 75–100% maximum heart rate), and strength exercises (2 h/session; 2 sessions/week; 8 in Borg RPE-CR10 scale). During the weeks of study, the rowers performed their training sessions without experiencing significant changes in their training routine. To this end, all training sessions were registered in a training diary during the intervention and the rowers replicated the same training sessions and training load prescribed by the team’s coach as explained above. In this regard, the rowers trained all together as a part of their seasonal preparation.

Before the nutritional intervention, an exhaustive nutritional anamnesis was performed by a registered dietitian to assess potential nutritional preferences, food intolerances and/or allergies, etc. During the entire nutritional intervention, nutritional intake and supplementation were prescribed by a registered dietitian. Nutritional plans were formulated to enhance health status and exercise performance of the participants by ensuring an appropriate energy intake (mean energy intake = 40.8 ± 3.5 kcal/kg body weight (BW)/day), following their respective training loads and energy expenditure, and following international consensus (Burke et al. 2019). Concretely, specific outdoor sessions (in the sea) were planned with 5–6 g carbohydrates (CHO) per kg BW, 2–2.2 g proteins (PRO), and 1.5–1.8 g fatty acids (FAT)/kg BW, respectively. Strength sessions were fueled accordingly (3–4 g CHO, 2.2–2.4 g PRO, and 1.5 g FAT/kg BW, respectively), and efforts sessions with 6–7 g CHO, 2–2.2 g PRO, and 1.25–1.5 g FAT/kg BW, respectively). Non-training sessions were adjusted to 2–2.5 g CHO, 2.4–2.5 g PRO and 1.25–1.5 g FAT/ kg BW. The day before each study visit, rowers were provided with an individualized and standardized meal plan aiming to guarantee glycogen load was successfully achieved (10 g CHO, 1.8 g PRO and 1.25 g FAT/kg BW).

The rowers were instructed to exclusively consume the designated supplement and refrain from using any other ergogenic aid/supplement. Moreover, participants were instructed to isolate daily supplement intake from toothpaste use and any other food and drinks, suggesting isolated intake of the supplement with water at the same time of the day (2 h before daily exercise and same time on rest day).

All study participants were thoroughly briefed on the research procedures and provided written informed consent. The study was conducted in compliance with the ethical principles outlined in the Declaration of Helsinki (2008) and its Fortaleza update (2013). Ethical approval was granted by the Human Research Ethics Committee at the University of Deusto, Bilbao, Spain (ETK-13/18–19).

### Nutritional supplementation randomization

As abovementioned, this is a randomized, double-blind, placebo-controlled, crossover study. Thus, rowers were randomized and allocated to one of the three groups by an independent investigator, and researchers and participants were not informed about the supplement intake in each condition. Each group corresponded to a predetermined supplement intake order (Fig. 1), and the supplements were: (i) Placebo group (PLAG); (ii) BR group (BRG); and (iii) BR + CIT group (BR-CITG). Supplementation intervention was performed in three consecutive weeks (i.e., 1 week per supplement intake condition). The amounts provided per day were: (i) 5 capsules of placebo (maltodextrin encapsulated by researchers in the same color capsules as the BR capsules) and 6 g of maltodextrin in powder; (ii) 5 capsules (500 mg) of BR (Lindens Health Nutrition®, Wakefield, UK) and 6 g of maltodextrin in powder; or, (iii) 5 capsules of BR (500 mg) and 6 g of CIT in powder (L-Citrulline powder, MyProtein, Manchester, UK) provided in a bag with a dispenser to each participant. Weekly, an independent investigator, who had prepared the capsules, gave each athlete his supplement and checked that the protocol was being carried out correctly according to the guidelines.

### Familiarization visit

Before starting the study, the rowers came to the laboratory for a familiarization session (Fig. 1) and measurement of basal conditions as explained below. Then, the rowers performed a 5-min warm-up at a subjective intensity of 5–6 on the CR10 Borg scale (Borg 1998) on a wind resistance brake rowing ergometer (Concept II, model D, Morrisville, VT, USA) equipped with a static seat, with the knees in semi-flexion and the length of the arms adapted to each rower. This position was maintained for all measurements performed on the rowing machine during the entire study. After the warm-up, the rowers performed the counter-movement jump (CMJ) test and a test for assessing their VO_2peak_. Both procedures are extensively detailed below.

After the familiarization session, the independent investigator in charge of randomization provided supplementation for the first week assigned randomly in groups, and the nutritional supplementation intervention started.

### Study visits for assessing dependent outcomes

The protocol described here was performed thrice, exactly following the same procedures, one after each week of supplementation (Fig. 1). The same procedure was replicated for each study visit, and the same standardized training and nutritional programs were followed. In addition, rowers were instructed not to perform any strenuous exercise 24-h before each study visit and to consume their individualized and standardized meal plan to guarantee glycogen load as detailed above.

For every visit, the rower attended the research center early in the morning after a 10–12 h overnight fasting period, avoiding stimulants 12 h before the study visit and drugs 24 h before. In addition, upon the rowers’ arrival, the researchers checked for compliance with rest (i.e., no exercise during the preceding 24 h) and the individualized nutritional plan.

Blood samples, from the antecubital vein, were obtained as detailed below. Then, the rower was weighed using a validated weight scale (InBody 570, In Body, Cerritos, CA, USA)., and heart rate and blood pressure were measured (M6 comfort, Omron, Hoofddorp, Netherlands) after lying, in the supine position, on a stretcher for 5 min. Subsequently, the rowers warmed up for 5-min at an intensity of 60% of the VO_2peak_ assessed in the familiarization visit (Fig. 1). Following the warm-up, the CMJ test was conducted. The second test consisted of the Wingate test, an all-out test in which the participant rowed with maximum drag at the highest possible intensity for 45 s.

Finally, after a passive recovery of 20 min, while the rower was sitting, a VO_2peak_ performance test was conducted. It is worth mentioning that the procedure for this test was identical to the VO_2peak_ test conducted during the familiarization session. As during the Wingate test, the gas exchange was continuously measured using a K5 system. All procedures are extensively detailed below, in their respective section.

### Blood samples and blood pressure assessments

After an overnight fast (10–12 h), blood samples were collected from the antecubital vein with the rowers seated in a comfortable position using a Vacutainer tube (BD Vacutainer, Beckton, Dickinson, and Company, Franklin Lanes NJ, USA) with gel and clot activator to obtain serum. Then, following standardized procedures were centrifuged (3000 revolutions per minute), aliquoted, and sent to refrigerated before analysis. Then, hemoglobin, hematocrit, leucocytes, urea, creatinine, total protein, albumin, prealbumin, gamma-glutamyl transferase, lactate dehydrogenase, glutamic pyruvic transaminase, glutamic-oxaloacetic transaminase, creatine kinase, c-reactive protein, testosterone, and cortisol were determined, and the testosterone-to-cortisol ratio calculated as follows: $\frac{{\text{Testosterone}}}{{\text{Cortisol}}}$.

Blood pressure was assessed twice, using an automatic monitor (M6 comfort, Omron, Hoofddorp, Netherlands), from the right arm while the rowers were resting in a sitting position.

### Countermovement jump test

After the warm-up, the CMJ test was conducted (Krishnan et al. 2017). A jumping mat (Chronojump-Boscosystem, Barcelona, Spain) was used (Pueo et al. 2017). Briefly, the rowers stand upright with hands on their hips, then, they performed a quick downward movement by flexing their hips and knees (to a 90-degree angle), and immediately after the downward movement, they explosively jump upward as high as possible. During the flight phase, the rowers should not bend the legs and at no time were the hands separated from the hips. The rowers made three attempts with a minimum of 30-s resting period between jumps. The best attempt was used for further analyses.

### Wingate test assessment

After the CMJ test, rowers were allowed to rest for a few minutes before eliciting the Wingate test. The test was conducted in the same row ergometer mentioned above, and all rowers performed the Wingate test in the same ergometer but with their individualized seat positioning (see Familiarization visit subsection). Finally, for all rowers, a maximum drag factor (aerodynamic resistance factor) was set.

Once the subject was ready, a researcher gave the start signal and the 45-s all-out Wingate anaerobic test started (Calbet et al. 1997). During the test, the rower was encouraged to give his maximum effort. Then, mean power (in Watts [W]), peak power and lowest power (maximum and minimum W yielded within the test, respectively), and stroke rate were recorded, and relative power (W relative to body weight) and fatigue index were computed as: $\frac{\mathrm{mean power}}{{{\text{rower}}}^{\mathrm{^{\prime}}}\mathrm{s body weigth}}$ and $\left(\frac{(\mathrm{peak power}-\mathrm{lowest power})}{\mathrm{peak power}}\right)\times 100$, respectively.

In addition, before the Wingate test starts, capillary lactate concentration (hereinafter blood *lactate concentration*) was determined from the ear using a digital lactate analyzer (Lactate Pro 2, Arkray, Barcelona, Spain) equipped with a blood lactate test strip (Lactate Pro 2, Arkray, Barcelona, Spain), heart rate was monitored using a chest strap sensor (Polar H10, Polar Electro Oy, Kempele, Finland), and capillary glucose concentration (hereinafter *blood glucose concentration*) was determined using a digital glucose analyzer (Exactive EQ Impulse, MicroTech Medical, Hangzhou, China) equipped with a blood glucose test strip (Exactive EQ Impulse, MicroTech Medical, Hangzhou, China). After the Wingate test, blood lactate concentrations were assessed 3- and 5-min post-exercise.

### Performance test assessment

After the Wingate test, a minimum of 20-min resting period was left to ensure that the rower recovered from the all-out exercise. The performance test started with an initial power of 135 W (with a drag factor of 160), increasing every 1 min by 25 W until volitional exhaustion. The rowers could select the cadence they felt most comfortable in and were encouraged by the evaluators throughout the test. The rowers were considered to have reached their peak performance and VO_2peak_ when at least two of the following criteria were met (Midgley et al. 2007): (i) a plateau in oxygen consumption, defined as an increase of ≤ 1.5 ml/kg/min in two consecutive workloads; (ii) respiratory exchange ratio > 1.15; and, (iii) maximum heart rate value > 95% of their predicted maximum heart rate value (using the 220 – age formula).

During the performance test, gas exchange was measured breath-by-breath using a K5 system. The K5 system was calibrated (both flow and gas analyzers) before each test strictly following the manufacturer’s recommendations. Heart rate was monitored during the test using a Polar H10 transmitter (Polar Electro, Lake Success, NY, USA). At the end of the test, peak power (watts), heart rate (maximum heart rate recorded), effort perception (CR10 Borg scale) (Borg 1998), blood lactate, and glucose concentrations were recorded.

### Statistical analysis

Results are presented as mean and standard deviation (SD) unless otherwise stated. All analyses were conducted using Statistical Package for Social Sciences (SPSS; v.25.0 for macOS, IBM SPSS Statistics, IBM Corporation, Chicago, IL, USA). All figures were created using GraphPad Prism software (v.9 for macOS, San Diego, California, USA). The significance level was set at *P* < 0.05.

Analysis of covariance (ANCOVA), with Bonferroni comparisons, was used for testing between study conditions and participants’ characteristics. Then, to test between-conditions differences in Wingate all-out test outcomes (i.e., lowest, maximum, mean, and relative power, fatigue index, and stroke rate), ANCOVA analysis, with Bonferroni comparisons was used. On the other hand, to further study the blood lactate levels concentrations during the Wingate test, a two-factor (*Time* × *Supplement*) repeated-measures analysis of variance (ANOVA), with Bonferroni comparisons was used. In addition, ANCOVA, with Bonferroni comparisons, was used for testing between-conditions differences in performance outcomes (i.e., peak power, VO_2peak_, maximum heart rate, post-performance test blood lactate, and blood glucose concentrations level, and Borg RPE-CR10 scale). Finally, to test between-conditions fasting blood samples, ANCOVA with Bonferroni comparisons was used.

## Results

A total of 20 men (29 ± 7 years old, 13 ± 6 rowing experience) participated in the study. Additional participants’ characteristics are presented in Table S1. Of note, body weight remained unaltered (*P* > 0.05) across study conditions (Table S1).

**Fig. 2:**
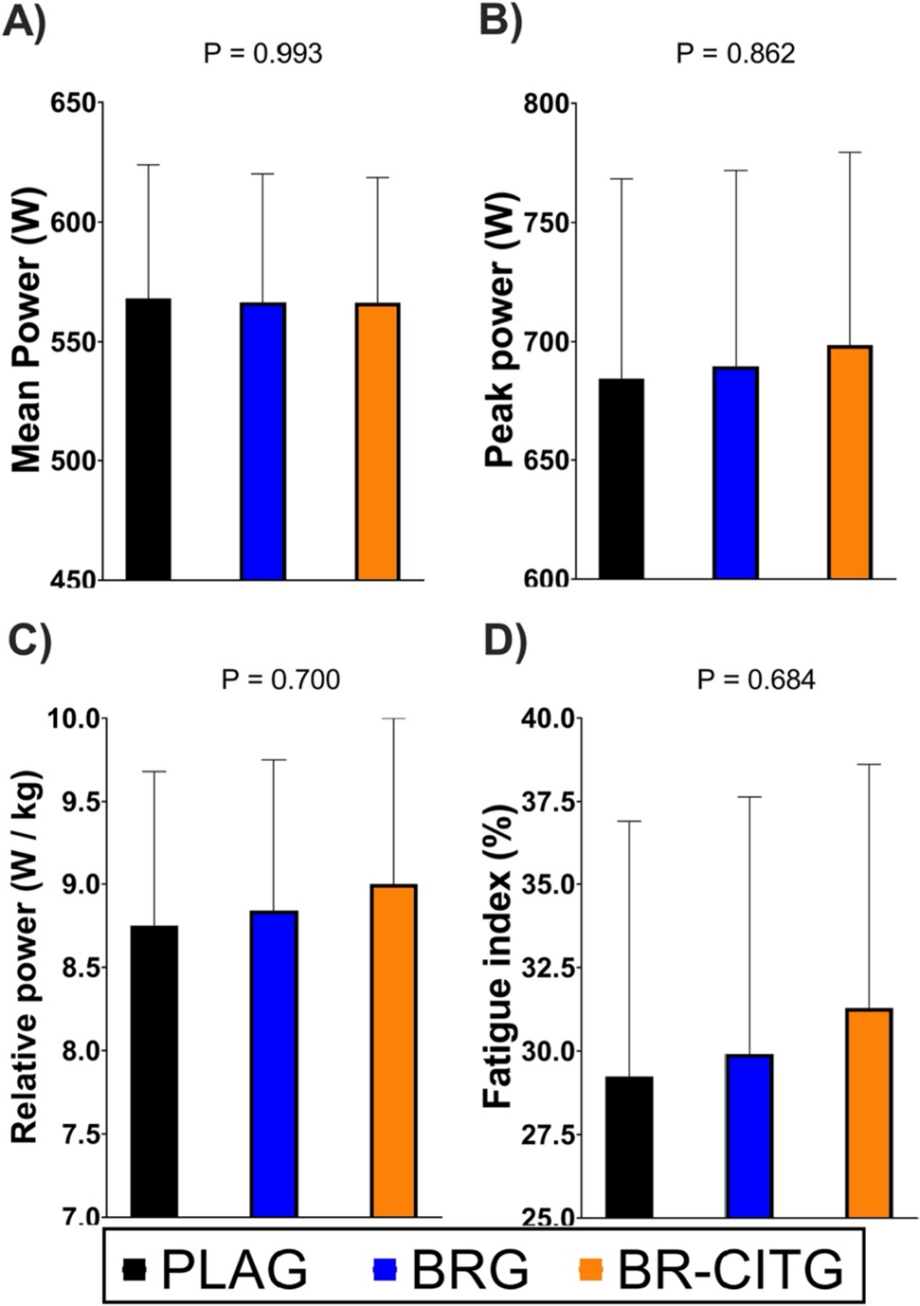
Wingate all-out test mean power (Panel **A**), peak power (Panel **B**), relative power (Panel **C**) and fatigue index (Panel **D**) performance results across study conditions. Notes: data are presented as mean and standard deviation. Mean and peak (i.e., peak power) were assessed within the Wingate test, while relative power was calculated as the mean power-to-body weight ratio, and the fatigue index as the ([peak power – the lowest power]/peak power) × 100. *PLAG* placebo supplement (maltodextrin) group. *BRG* nitrate-rich beetroot extract supplement group. *BR-CITG* BR extract plus citrulline (CIT) supplement group. *P* values derived from analysis of covariance (ANCOVA; *n* = 20) to examine between-conditions adjusted mean differences

**Fig. 3:**
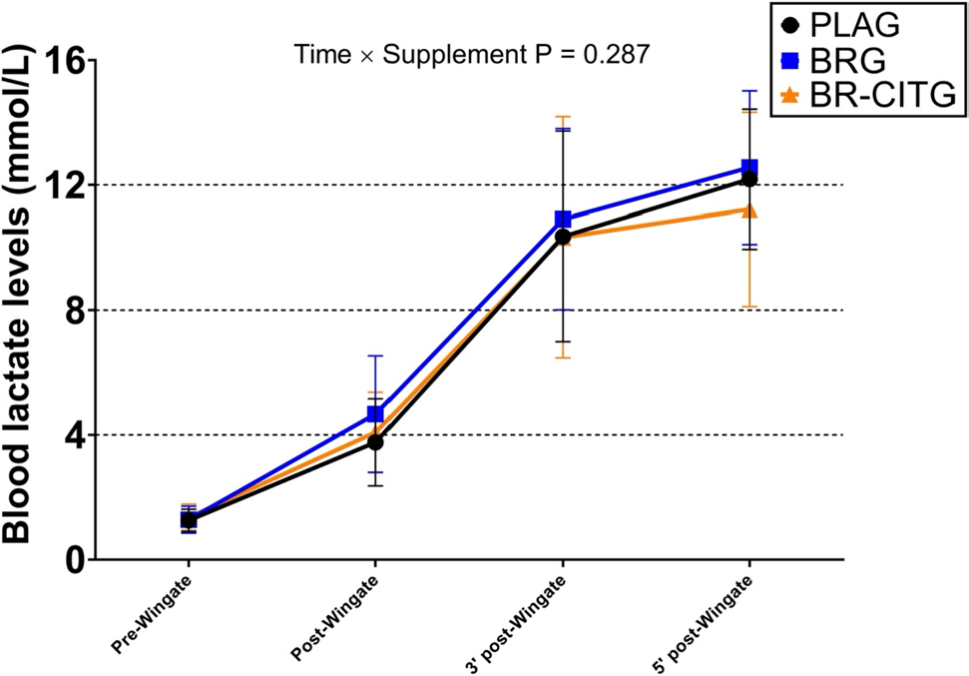
Capillary blood lactate concentration obtained across time and supplement conditions within the Wingate test. Notes: Pre-, Post-, 3ʹ-post and 5ʹ post-Wingate periods correspond to each period in which the lactate concentration was measured. Data are presented as mean and standard deviation. *PLAG* placebo supplement (maltodextrin) group, *BRG* nitrate-rich beetroot extract supplement group, *BR-CITG* BR extract plus citrulline (CIT) supplement group. *P* value from two-factor (Time × Supplement) repeated-measures analysis of variance (ANOVA; *n* = 20) comparisons

ANCOVA analyses showed no between-conditions differences in peak power (Fig. 2A), mean power (Fig. 2B), relative power (Fig. 2C), and fatigue index (Fig. 2D) outputs from the Wingate all-out test. In addition, repeated-measures ANOVA showed no *Time* × *Supplement* interaction effect at different points of sampling (pre-Wingate, post-Wingate, and 3-min and 5-min post-Wingate; Fig. 3). Blood lactate concentration patterns were similar regardless of the supplement intake, and all showed a significant *Time* effect (all *P* < 0.001). Bonferroni post hoc analyses showed differences between all period comparisons for PLAG (all *P* < 0.05), and BR (all *P* < 0.05), while for BR-CITG differences were observed for all period comparisons but 3rd vs. 4th period (*P* = 0.211). Additional outcomes related to the Wingate test are presented in Table S2.

**Fig. 4:**
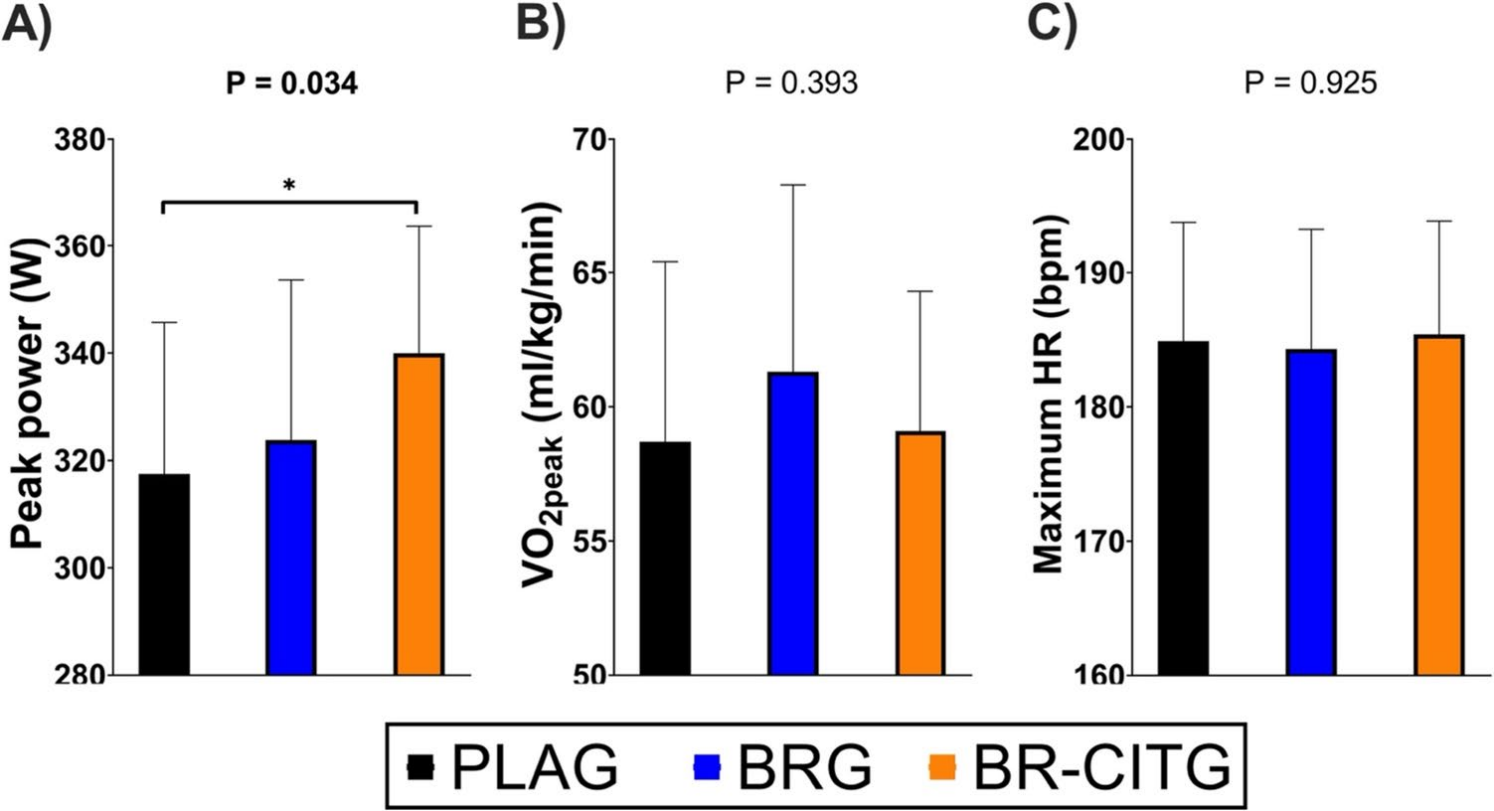
Performance test peak power (Panel **A**), whole-body peak oxygen uptake (VO_2peak_, Panel **B**), and maximum heart rate (HR, Panel **C**) results across study conditions. Notes: data are presented as mean and standard deviation. All outcomes were assessed within the performance test (i.e., effort test until volitional exhaustion). *PLAG* placebo supplement (maltodextrin) group, *BRG* nitrate-rich beetroot extract supplement group, *BR-CITG* BR extract plus citrulline (CIT) supplement group. *P* values derived from analysis of covariance (ANCOVA; *n* = 20) to examine between-conditions adjusted mean differences

ANCOVA analyses showed between-conditions differences in peak power (Fig. 4A) output from the performance test. However, no between-conditions differences were observed in VO_2peak_ (Fig. 4B) and maximum heart rate (Fig. 4C). Finally, Bonferroni post hoc analyses showed differences between PLAG and BR-CITG (*P* = 0.036; Fig. 4A). Additional outcomes related to the performance test are presented in Table S3.

**Fig. 5:**
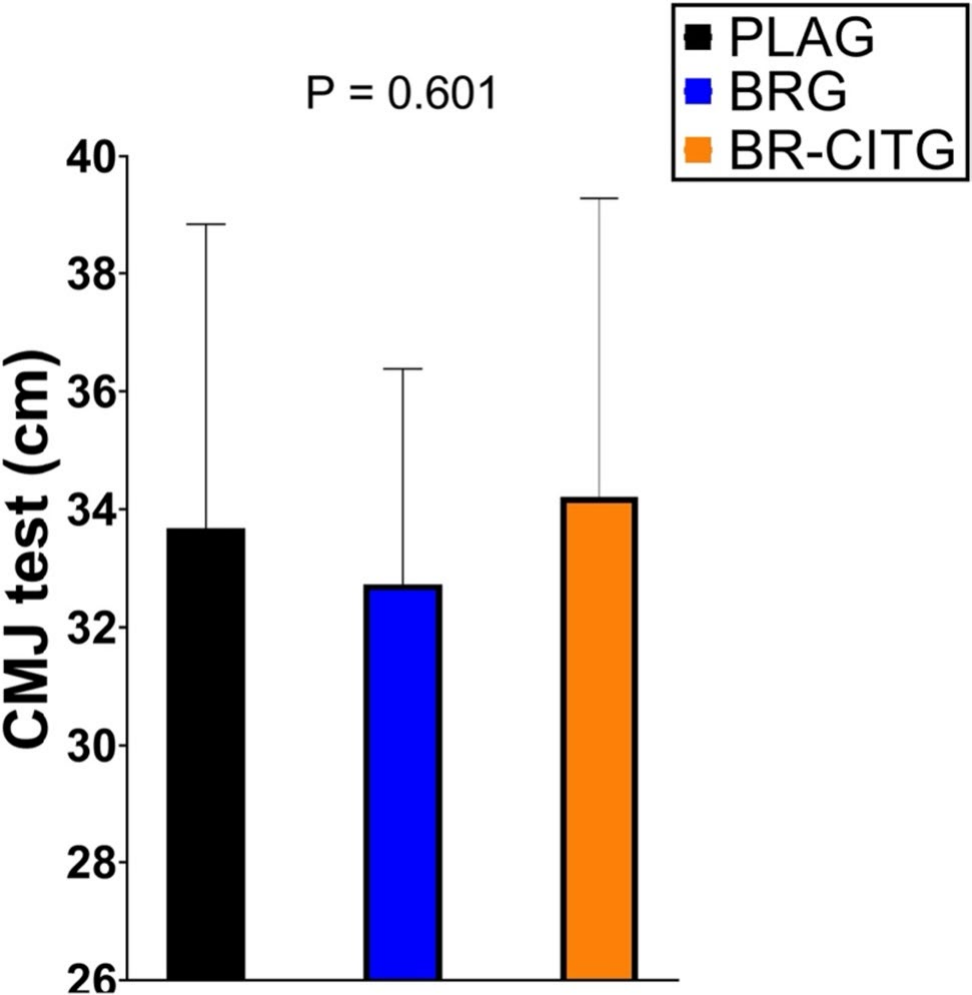
CMJ jump test height (cm) results across study conditions. Data are presented as mean and standard deviation. Notes: Data are presented as mean and standard deviation. *PLAG* placebo supplement (maltodextrin) group. *BRG* nitrate-rich beetroot extract supplement group. *BR-CITG* BR extract plus citrulline (CIT) supplement group. *P* values derived from analysis of covariance (ANCOVA; *n* = 20) to examine between-conditions adjusted mean differences

ANCOVA analyses showed no between-conditions differences in maximum height (cm) achieved in the CMJ jumping test (Fig. 5).

Finally, no between-conditions differences were observed in blood samples (all *P* > 0.200), as all blood outcomes remained unaltered between study conditions (Table S4).

## Discussion

This study aimed to assess the impact of short-term chronic (1 week) supplementation with oral co-ingestion of 6 g of CIT and 3.5 g of BR extract (500 mg or 8 mmol NO_3_^−^) per day on maximal strength (CMJ jump test), high-intensity power (mean, relative and peak power, and fatigue index in Wingate test), and aerobic performance (by analyzing power output, VO_2 peak_, lactate clearance and heart rate) in elite male rowers, compared with BR extract taken alone and PLA. The results showed no between-conditions (PLAG, BRG, and BR-CITG supplementation intervention) differences in Wingate test-related outcomes. However, 5-min post-Wingate test blood lactate concentration levels showed a significant difference in BR-CITG condition compared to BRG and PLAG conditions, thus suggesting a better post-exercise lactate clearance. Furthermore, differences were found in peak power during the aerobic performance test in BR-CITG compared to PLAG. Nonetheless, other related physiological parameters such as VO_2peak_ and maximum heart rate showed no differences. No other differences were observed in CMJ test-related outcomes and fasting blood circulating markers. Our results might confirm the proposed synergic effects of BR-CITG supplementation.

NO is an important bioactive molecule that has been related to improved oxygen (O_2_) delivery by impacting outcomes such as VO_2_ max, exercise economy, and fractional utilization of the VO_2_ max (Jones 2014b). As NO_3_^−^ and CIT serve as precursors of endogenous NO production (Lundberg et al. 2008; Milton-Laskibar et al. 2021), supplements containing NO_3_^−^ such as BR extract and CIT abundant supplements like citrulline-malate have been studied to understand potential effects on O_2_ delivery. Larsen et al. (2007) studied for the first time the impact of 3 days of sodium nitrate supplementation (0.1 mmol/kg body weight) in VO_2 max_ and no effects were found, but surprisingly a reduction of 5% in O_2_ cost at submaximal intensity (80% VO_2 max_) was observed. Subsequent studies using BR supplementation confirmed similar findings (Bailey et al. 2009, 2010; Vanhatalo et al. 2010; Cermak et al. 2012; Wylie et al. 2013a) to those observed by Larsen et al. (2007), although some positive changes have been also observed in VO_2 max_ (Larsen et al. 2010). To date, a direct effect of any form of NO_3_^−^ supplementation on VO_2 max_ is elusive, and more studies are needed. However, NO_3_^−^ supplementation benefits on exercise economy seem to be ineludible (Jones 2014b). On the other hand, supplementation of both BR and CIT separately has shown improvements in aerobic performance. Hoon et al. (2014) observed improvements in aerobic power, defined as time to complete a 2000 m test in highly trained rowers after ingesting 140 ml BR juice (8.4 mmol NO_3_^−^) 2 h before exercise. Their results were later confirmed in a study carried out on trained cyclists, as the authors observed that BR supplementation 2.5 h before the exercise reduced the time necessary to complete the 4 km time trial (Lausch et al. 2017). Interestingly, in the study by Lausch et al. (2017), the BR supplementation was lower (6.2 mmol NO_3_^−^) than in the Hoon et al. (2014) study. Beyond acute supplementation, NO_3_^−^ chronic intake elicits potential interest due to the evidenced muscle’s capacity to accumulate, transfer and metabolize NO_3_^−^ and nitrite (NO_2_^−^), and its key role in whole-body NO pool maintenance (Nyakayiru et al. 2020; Piknova et al. 2022). In this regard, Cermak et al. (2012) and Rokkedal-Lausch et al. (2019) found improvements in 10-km cycling time trial in trained and well-trained cyclists after 7 days of supplementation with 12.4 and 8.8 mmol BR juice, respectively. Similar results have been reported following short-term chronic CIT supplementation. For instance, improvements in time to complete a 4 km cycling time trial were shown after ingesting 2.4 g of CIT for 7 days (Suzuki et al. 2016). Chronic intake (daily intake during 9 consecutive weeks) of 3 g/day of CIT combined with 2.1 g/day of BR extract led to improvements in estimated VO_2 max_ (using the following formula: 22.351 × Distance covered (km) in Cooper test – 11.288) and aerobic power performance of experienced triathletes when compared to placebo and BR extract conditions alone (Burgos et al. 2021). In the current study, the obtained peak power in the incremental aerobic exercise protocol test showed significant differences when comparing co-ingestion of CITG and BRG to PLAG, but we did not observe differences vs. the BRG condition. These benefits, however, were not accompanied by improved VO_2 peak_ or heart rate responses. Although more data are needed to confirm the effects observed in our study and to better understand the physiological mechanisms behind them, results might suggest that short-term chronic (i.e., 7 days) co-supplementation of CIT and BR supplements could improve peak power elicited during an aerobic performance test in elite male rowers.

Previous literature has described that NO may have a positive effect on blood vessel dilation and blood flow increment specifically in type II muscle fibers (Ferguson et al. 2013; Jones et al. 2016). BR juice can improve the release and re-uptake of calcium in the sarcoplasmic reticulum, thus improving muscle contractile capacities (Hernández et al. 2012). Therefore, NO_3_^−^ supplements intake could potentially offer positive effects in high-intensity efforts where the ability to deliver and maintain high peak power is determinant, such as rowing (Bell et al. 1989). Wylie et al. (2013b) found, in recreational team-sport players, improvements in 6 s sprints after supplementing 8.4 mmol NO_3_^−^ for 8 days, but no other benefits were reported in sprints lasting between 30 and 60 s. However, using similar supplementation (8 mmol NO_3_^−^ for 7 days), benefits in 60 s sprint have been found in active men (Rowland et al. 2023). Although other benefits have been reported in efforts lasting 30–60 s such as after a Wingate test (i.e., 45 s), these studies used acute intake of NO_3_^−^ (Dominguez et al. 2017b; Jodra et al. 2020). However, when combined supplementation with BR extract and CIT was given to triathletes for 9 weeks, improvements in 1 min maximal abdominal test were shown, suggesting potential synergic effects between both supplements (Burgos et al. 2021). CIT supplementation can stimulate muscle protein synthesis and, therefore, could potentially improve muscle strength (Goron et al. 2019). Nonetheless, the literature addressing the unbiased effect of CIT supplementation on sport performance is scarce. In our present study, the combined supplementation strategy showed no effect (Fig. 2) on 45 s Wingate test performance and its related outcomes (peak power, mean power, relative power, and fatigue index), suggesting 1 week of supplementation is not enough to generate hypothesized muscle adaptations. Interestingly, we showed a better blood lactate concentration clearance in the BR-CITG condition compared to BRG and PLAG conditions after 5 min post-Wingate test (Fig. 3). As lactate clearance relies on oxidative capacity, it is plausible that the reported effects of supplementation on aerobic metabolism could positively impact on athletes’ oxidative capacity and therefore be an indirect sign of improved aerobic performance. Some studies have also reported lower blood lactate concentrations after CIT supplementation (Rhim et al. 2020; Divito et al. 2022), but the literature regarding BR supplements remains unclear. In fact, potential mechanisms related to lower blood lactate values after CIT supplementation might be related to enhanced aerobic pathway, as reported for instance by higher levels of plasma lactate dehydrogenase enzyme (Rhim et al. 2020), which could be in accordance to previously identified effects of NO-related supplements such as BR. In addition, our study showed no improvements in CMJ jump between conditions (Table S1), a finding that contrasts with improvements found in horizontal jump after 9 weeks of the same combined supplementation (Burgos et al. 2021), and may suggest the need for longer than 7 days supplementation periods to improve strength and high peak power outcomes in elite rowers. Therefore, these results refuted the proposed hypothesis of a complementary way of improving muscle strength through a better excitation–contraction coupling after 6 g CIT and 3.5 g BR extract (500 mg or 8 mmol NO_3_^−^) supplementation. As it has been hypothesized that elite athletes are potentially less sensitive to improvements after BR supplementation (Dominguez et al. 2017a), it is plausible that quantities required to show beneficial effects might be higher than 8 mmol NO_3_^−^/day or should be related to body weight or fat-free mass, to individualize the supplement dose to the athlete.

Concerning biochemical biomarkers, we showed no differences between conditions after 1 week of supplement intake. Although some literature has studied the influence of CIT and BR supplementation alone on recovery status through hormones and functional tests (da Silva et al. 2017; Garnacho-Castaño et al. 2020; Daab et al. 2021), their effect remains unclear and more studies are needed. However, 9 weeks of BR + CIT supplementation showed improvements in the testosterone-to-cortisol ratio accompanied by enhanced aerobic performance test (Burgos et al. 2022). Considering all together, our study reinforces the idea to study longer periods of CIT and BR combined intake in elite rowers.

The impossibility to measure blood NO_3_^−^ and NO_2_^−^ concentrations in participants after ingesting supplements elicits a potential limitation that, however, it can be less determinant considering the chronic supplementation protocol used in this study. Methodologically, the absence of a CIT-only supplementation group can be considered a limitation to be noted, although CIT alone did not show any benefits compared to BR-only and BR + CIT supplementation in previous studies (Burgos et al. 2021, 2022). Moreover, muscle and strength-related effects could be taken as speculative as only performance tests were performed and no muscle samples (e.g., biopsies) were obtained for analyses. Finally, considering the impact of oral and gut microbiota and environment in NO_3_^−^ and NO_2_^−^ metabolism that can lead to different results (González-Soltero et al. 2020), the lack of rowers’ microbiota samples could represent a limitation. Nevertheless, the sample was composed of high-level rowers, which is a real strength of this study, due to the difficulty to access to this kind of athletes. Moreover, the design of the study and performance tests included in the study should be also acknowledged.

Future research should continue to study the combination of NO-related supplements in the long term to better understand their role in muscle NO_3_^−^ and NO_2_^−^ storage and related physiological and metabolic mechanisms. In addition, the potential synergic effects of supplements using compatible pathways in different performance outcomes could be of interest. Moreover, interventions lasting between 1 and 9 weeks should be addressed to be able to design higher efficacy supplementation protocols. Beyond measuring performance, mechanisms which by NO could impact oxidative and glycolytic metabolism, and muscle contractile function should be also studied in detail as this remains partially unknown yet. Finally, the impact of exogenously stimulated NO production in female athletes remains to be elicited to date, and therefore, it should be widely studied.

## Practical implications

Combining supplements with similar physiological and metabolic mechanisms is interesting to optimize supplementation protocols in athletes regardless of their training/competition level. This study showed that even elite rowers find certain (although slightly) performance improvements by BR + CIT supplementation for 1 week.

## Conclusion

The daily combination of 3.5 g of BR extract plus 6 g of CIT supplementation for 7 days seems to improve blood lactate clearance (oxidative capacity) compared to the PLA after the completion of a Wingate test, and the peak power after an aerobic performance test in a sample of elite rowers. No further improvements in maximal strength, high-intensity power and peak stroke power, and other aerobic performance-related outcomes were observed between supplements conditions. Our results may suggest that more than 7 days are necessary to better understand the potential synergic effects of BR + CIT supplements on sports performance.

### Supplementary Information

Below is the link to the electronic supplementary material.

Supplementary file1 (DOCX 20 KB)

## Data Availability

The datasets generated during and/or analysed during the current study are available from the corresponding author on reasonable request.
