# Supplementary material for: Impact of a short-term nitrate and citrulline co-supplementation on sport performance in elite rowers: a randomized, double-blind, placebo-controlled crossover trial
Source: Eur J Appl Physiol. 2024 Feb 10;124(6):1911–23. doi: 10.1007/s00421-024-05415-4 (PMC11129974; doi:10.1007/s00421-024-05415-4)
Supplement: Supplementary file 1 — Supplementary file1 (DOCX 20 KB) [file 421_2024_5415_MOESM1_ESM.docx]

*Title of the article*

**Impact of a short-term Nitrate and Citrulline co-supplementation on sport performance in elite rowers: A randomized, double-blind, placebo-controlled crossover trial.**

*Submission type: Original article*

**Authors**

Aitor Viribay^1,2^, Juan MA Alcantara^3,4,5^, Iker López^6^, Juan Mielgo-Ayuso^7*^, Arkaitz Castañeda-Babarro^8^

**Affiliations**

^1^Glut4Science, Physiology, Nutrition and Sport, Vitoria-Gasteiz 01004, Spain; [aitor@glut4science.com](mailto:aitor@glut4science.com)

^2^ Institute of Biomedicine (IBIOMED), University of Leon, 24071 Leon, Spain.

^3^ Institute for Sustainability & Food Chain Innovation, Department of Health Sciences, Public University of Navarre, Pamplona, Spain.

^4^ Navarra Institute for Health Research, IdiSNA, Pamplona, Spain.

^5^ Centro de Investigación Biomédica en Red Fisiopatología de la Obesidad y Nutrición (CIBERobn), Instituto de Salud Carlos III, 28029 Madrid, Spain. [juanmanuel.alcantara@unavarra.es](mailto:juanmanuel.alcantara@unavarra.es)

^6^ Kirolene, San Ignacio Auzunea Etxetaldea 5, Durango 48200, Spain.

^7^ Department of Health Sciences, Faculty of Health Sciences, University of Burgos, 09001 Burgos, Spain. [jfmielgo@ubu.es](mailto:jfmielgo@ubu.es)

^8^ Health, Physical Activity, and Sports Science Laboratory, Department of Physical Activity and Sports, Faculty of Education and Sport, University of Deusto, 48007 Bizkaia, Spain. [arkaitz.castaneda@deusto.es](mailto:arkaitz.castaneda@deusto.es)

**Correspondence to**

Dr. Juan Mielgo Ayuso

Department of Health Sciences, Faculty of Health Sciences, University of Burgos, 09001 Burgos, Spain.

[jfmielgo@ubu.es](mailto:jfmielgo@ubu.es)

**Supplementaty Material**

**Table S1.** Participants' characteristics across study conditions.

|  | **PLA** | **BR** | **BR+CIT** | **P** |
| --- | --- | --- | --- | --- |
| Body weight (kg) | 78.39 ± 7.50 | 78.27 ± 7.73 | 77.90 ± 7.67 | 0.978 |
| Resting heart rate (bpm) | 59.50 ± 9.74 | 60.65 ± 10.78 | 57.30 ± 10.45 | 0.584 |
| Systolic blood pressure (mmHg) | 149.45 ± 9.18 | 148.15 ± 12.18 | 144.30 ± 13.61 | 0.364 |
| Diastolic blood pressure (mmHg) | 81.10 ± 6.64 | 81.80 ± 6.83 | 78.60 ± 6.57 | 0.289 |

Notes: Data are presented as mean ± standard deviation (SD). P value from ANCOVA analyses.

**Table S2**. Wingate test.

|  | **PLA** | **BR** | **BR+CIT** | **P** |
| --- | --- | --- | --- | --- |
| Lowest power (W) | 479.95 ± 43.97 | 479.30 ± 45.38 | 476.25 ± 42.49 | 0.960 |
| Stroke rate (spm) | 42.30 ± 4.64 | 41.77 ± 4.32 | 41.86 ± 7.78 | 0.953 |

Notes: Data are presented as mean ± standard deviation (SD). P value from ANCOVA analyses.

**Table S3.** Outcomes assessed after the performance test.

|  | **PLA** | **BR** | **BR+CIT** | **P** |
| --- | --- | --- | --- | --- |
| Blood lactate concentration (mg/dl) | 7.74 ± 3.54 | 6.30 ± 2.00 | 6.99 ± 2.21 | 0.243 |
| Blood glucose concentration (mg/dl) | 87.30 ± 16.12 | 87.75 ± 13.57 | 92.55 ± 18.49 | 0.528 |
| Borg RPE-CR10 scale | 9.13 ± 0.86 | 9.03 ± 0.68 | 9.18 ± 1.05 | 0.860 |

Notes: Data are presented as mean ± standard deviation (SD). P value from ANCOVA analyses.

**Table S4.** Blood (fasting) samples.

|  | **PLA** | **BR** | **BR+CIT** | **P** |
| --- | --- | --- | --- | --- |
| Hemoglobin (g/dl) | 14.97 ± 0.79 | 14.85 ± 0.78 | 14.88 ± 0.79 | 0.892 |
| Hematocrit (%) | 45.81 ± 2.92 | 45.14 ± 2.01 | 45.16 ± 2.59 | 0.640 |
| Leucocytes (10^9/l) | 9.30 ± 3.06 | 8.76 ± 2.62 | 7.98 ± 2.19 | 0.291 |
| Urea (mg/dl) | 38.55 ± 5.91 | 37.65 ± 8.63 | 37.10 ± 6.85 | 0.815 |
| Creatinine (mg/dl) | 1.19 ± 0.18 | 1.15 ± 0.14 | 1.14 ± 0.15 | 0.619 |
| Total protein (g/dl) | 7.51 ± 0.33 | 7.45 ± 0.32 | 7.54 ± 0.34 | 0.700 |
| Albumin (g/dl) | 5.08 ± 0.21 | 5.05 ± 0.20 | 5.06 ± 0.26 | 0.923 |
| Prealbumin (mg/dl) | 28.50 ± 7.88 | 25.90 ± 4.27 | 26.05 ± 4.44 | 0.287 |
| GPT (UI/L) | 24.95 ± 24.39 | 21.25 ± 12.28 | 20.20 ± 9.78 | 0.644 |
| GOT (UI/L) | 24.85 ± 6.87 | 25.60 ± 9.24 | 23.35 ± 8.57 | 0.684 |
| GGT (U/L) | 50.15 ± 167.58 | 40.30 ± 118.15 | 32.40 ± 88.48 | 0.909 |
| Creatine kinase (mg/dl) | 267.5 ± 154.6 | 274.9 ± 188.0 | 203.4 ± 103.3 | 0.273 |
| LDH (mg/dl) | 204.7 ± 43.4 | 200.6 ± 40.9 | 183.8 ± 31.4 | 0.206 |
| C-reactive protein (mg/dl) | 0.04 ± 0.04 | 0.04 ± 0.03 | 0.03 ± 0.02 | 0.825 |
| Testosterone (ng/dl) | 4.80 ± 1.88 | 5.28 ± 1.86 | 5.53 ± 2.28 | 0.517 |
| Cortisol (mcg/dl) | 15.78 ± 6.68 | 16.43 ± 4.53 | 15.03 ± 4.66 | 0.717 |
| Testosterone-to-Cortisol ratio | 0.37 ± 0.25 | 0.36 ± 0.20 | 0.41 ± 0.25 | 0.832 |

Notes: Data are presented as mean ± standard deviation (SD). P value from ANCOVA analyses. GGT: gamma-glutamyl transferase. LDH: lactate dehydrogenase. GPT: glutamic pyruvic transaminase. GOT: glutamic-oxaloacetic transaminase
